# Modern Methods for Detection of Fentanyl and Its Analogues: A Comprehensive Review of Technologies and Applications

**DOI:** 10.3390/molecules30173577

**Published:** 2025-08-31

**Authors:** Ewelina Bojarska, Wojciech Zajaczkowski, Elwira Furtak, Maksymilian Stela, Leslaw Gorniak, Marcin Podogrocki, Michal Bijak

**Affiliations:** 1 Department of Protection Against Contamination, Military Institute of Chemistry and Radiometry, Antoniego Chrusciela “Montera” 105, 00-910 Warsaw, Poland; e.bojarska@wichir.waw.pl (E.B.); e.furtak@wichir.waw.pl (E.F.); 2 Biohazard Prevention Centre, Faculty of Biology and Environmental Protection, University of Lodz, Pomorska 141/143, 90-236 Lodz, Poland; wojciech.zajaczkowski@biol.uni.lodz.pl (W.Z.); maksymilian.stela@biol.uni.lodz.pl (M.S.); leslaw.gorniak@biol.uni.lodz.pl (L.G.); marcin.podogrocki@biol.lodz.pl (M.P.)

**Keywords:** fentanyl detection, field-deployable sensors, Raman/SERS, FTIR, GC–MS, LC–MS–MS, electrochemical sensors, aptamer biosensors, CBRN

## Abstract

Fentanyl and its analogues represent a severe threat due to their extreme potency and increasing prevalence in illicit drug supplies. Even trace amounts (on the order of a couple of milligrams) can be lethal, contributing to a surge in opioid overdose deaths worldwide. Beyond the public health crisis, fentanyl has emerged as a security concern, with the potential for deliberate use as a chemical agent in CBRN scenarios. This underscores the critical need for rapid and accurate detection methods that can be deployed by security forces and first responders. Modern technology offers a range of solutions—from portable mass spectrometers and spectroscopic devices to electrochemical sensors and immunoassay kits—that enable on-site identification of fentanyl and its analogues. This review provides a comprehensive overview of detection techniques, examining their capabilities and applications in law enforcement, border control, and CBRN incident response. We highlight how integration of advanced sensors with machine learning is enhancing detection accuracy in complex field environments. Challenges such as operational constraints and the ever-evolving variety of fentanyl analogues are discussed, and future directions are recommended to improve field-deployable detection tools for safety and security applications.

## 1. Introduction

Fentanyl is a strong synthetic opioid originally developed for clinical use in anesthesiology and the treatment of severe pain. Its potency is approximately 50 to 100 times that of morphine and approximately 50 times that of heroin. A lethal dose for an average adult is estimated to be as low as 2 milligrams [1]. In medical settings, fentanyl is administered with precision and monitoring, but its extraordinary potency entails a narrow therapeutic window and significant risk of overdose.

The emergence of fentanyl analogues, which are chemical variants such as sufentanil, alfentanil, and carfentanil, has been identified as a significant risk factor for the occurrence of undesirable effects. Notably, carfentanil, in particular, has been estimated to possess a potency 10,000 times greater than morphine. Its primary application is in the field of veterinary anesthesia [2].

**Figure 1:**
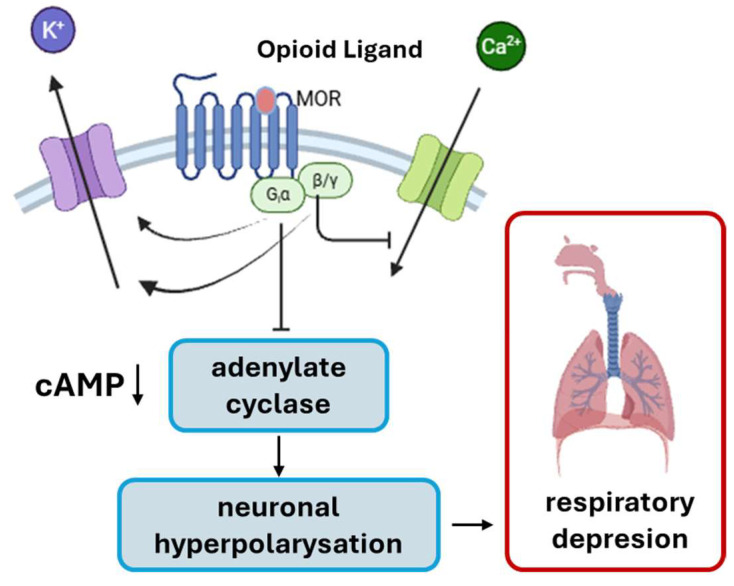
Schematic representation illustrates the mechanism by which fentanyl induces respiratory failure.

Fentanyl and its analogues are drugs that reduce pain. However, overdosing on opioids can cause severe breathing issues that may be fatal. When fentanyl binds to the μ-opioid receptor (a G protein-coupled receptor), it induces a conformational change that promotes coupling to the heterotrimeric Gα_i/Gα_oβγ complex. The activated Gα_i/_o subunit then inhibits adenylate cyclase activity, reducing intracellular cAMP concentrations and attenuating downstream cAMP-dependent signaling pathways. At the same time, Gα_i/_o activation opens G protein-gated inwardly rectifying K^+^ channels (GIRKs), facilitating potassium efflux and producing membrane hyperpolarization. In parallel, βγ heterodimers inhibit voltage-gated Ca^2+^ channels (N-type and P/Q-type), thereby reducing calcium influx. These actions together reduce presynaptic neurotransmitter release, dampen postsynaptic excitability and ultimately suppress neuronal activity, including that of respiratory control neurons in the brainstem [3,4]. Figure 1 provides a schematic overview of the signaling mechanisms triggered by fentanyl binding to the μ-opioid receptor.

While several analogues have legitimate clinical applications, many others are illegally synthesized in clandestine laboratories and distributed as illicit drugs, often mixed with heroin, cocaine, methamphetamine, or counterfeit prescription pills. The presence of fentanyl in such mixtures often remains undetected by users, leading to an increased risk of unintentional overdose.

This phenomenon constitutes the basis of the contemporary opioid epidemic. In recent years, a global decline in overdose deaths has been observed. In the United States, both age-adjusted rates and the number of deaths involving synthetic opioids have shown a sustained year-on-year decrease. Preliminary data for 2024 indicate a further ~27% reduction in opioid-related fatalities, with most states reporting declines. In Europe, mortality remains markedly lower than in the U.S. but rose slightly in 2023 to 7459 deaths (7145 in 2022), with opioids implicated in approximately 70% of cases and fentanyl-related deaths remaining stable. In Asia, systematic mortality data remain scarce; however, UNODC reports continued expansion of synthetic drug markets and emerging detections of potent synthetic opioids, although no surge comparable to North America’s fentanyl crisis has been recorded. Despite the recent global downtrend in overdose mortality, synthetic opioids—and fentanyl in particular—continue to pose a significant potential threat, including their possible misuse in terrorist attacks [5,6,7,8]. The crisis’s implications extend beyond public health, posing a growing threat to law enforcement and national security. The high potency and portability of fentanyl in small quantities render it an optimal contraband substance. Furthermore, there are concerns regarding its potential for misuse in the context of chemical attacks. A notable example of this phenomenon is the 2002 hostage crisis at a Moscow theatre, where a fentanyl derivative (carfentanil and remifentanil) was used as an incapacitating agent, resulting in the deaths of over 120 civilians [9,10,11].

The increase in the illegal distribution of fentanyl has made effective detection tools more necessary than ever. Law enforcement, customs officers, paramedics, healthcare professionals, and harm reduction organizations require fast, sensitive, and reliable methods to identify fentanyl and its analogues. The Scientific Working Group for the Analysis of Seized Drugs (SWGDRUG) provides widely adopted guidance for classifying analytical techniques and structuring identification workflows. In this framework, Category A methods deliver the highest structural specificity (e.g., mass spectrometry, infrared or Raman spectroscopy), Category B provide intermediate selectivity (e.g., chromatographic or mobility-based separations), and Category C comprise presumptive screening assays (e.g., color tests, immunoassays). The categories reflect discriminating power and robustness in complex matrices and are intended to be used complementarily rather than as a simple hierarchy. Throughout this review we follow the SWGDRUG scheme, emphasizing orthogonal combinations—ideally including at least one Category A method where practicable—to ensure reliable identification of fentanyl and its analogues across operational contexts [12].

The increase in the illegal distribution of fentanyl has made effective detection tools more necessary than ever. Law enforcement, customs officers, paramedics, healthcare professionals, and harm reduction organizations need fast, sensitive, and reliable methods to identify fentanyl and its analogues. In the context of drug identification, techniques can be divided into three categories: Category A (Mass Spectrometry, Infrared Spectroscopy, Nuclear Magnetic Resonance Spectroscopy, and Raman Spectroscopy), Category B (Capillary Electrophoresis, Gas Chromatography, Ion Mobility Spectrometry, Liquid Chromatography Microcrystalline Tests, Supercritical Fluid Chromatography, and Thin-Layer Chromatography) and Category C (Color Tests, Fluorescence Spectroscopy, and Immunoassay).

Infrared spectroscopy Category A techniques are highly specific and can uniquely identify a substance based on its molecular structure or chemical composition. Regulatory bodies such as the UNODC (Office on Drugs and Crime) and SWGDRUG (Scientific Working Group for the Analysis of Seized Drugs) often require or recommend the use of these techniques.

Conventional laboratory techniques such as gas chromatography coupled with mass spectrometry (GC–MS) and liquid chromatography coupled with mass spectrometry (LC–MS) are highly sensitive and specific, and are therefore considered the gold standard for confirmatory testing [9]. However, they are not well suited to real-time field deployment due to limitations such as the size of the equipment and the requirements for sample preparation.

The demand for devices that can be used in the field has led to the development of various portable detection technologies. These include preliminary tools such as colorimetric and lateral-flow immunoassays (LFI), as well as more advanced systems such as handheld Raman and FTIR (Fourier-transform infrared spectroscopy) spectrometers, ion mobility spectrometers (IMS), and portable mass spectrometers, including DART–MS and HPMS [13,14]. While color tests are inexpensive and have gained wide popularity, they are characterized by limited specificity and a high rate of false-positive or -negative results, especially at low fentanyl concentrations. LFI boasts superior selectivity, exhibiting minimal cross-reactivity with prevalent substances such as cocaine or methamphetamine. However, its accuracy is susceptible to environmental influences and subjective interpretation of weak test lines [15].

A multi-layered approach that integrates screening and confirmatory technologies has demonstrated considerable promise for field detection. For instance, IMS can generate an initial alarm, subsequently followed by confirmatory tests employing portable GC–MS or DART–MS systems. This strategy enhances the certainty of detection and reduces the occurrence of false alarms, particularly in the context of complex mixtures. The integration of artificial intelligence and machine learning with spectral analysis represents a promising development that could further enhance the accuracy and efficiency of on-site identification, particularly for novel or low-concentration fentanyl analogues [16].

This review methodically appraises contemporary fentanyl detection methodologies, emphasizing their practical performance, strengths, and limitations across a range of field scenarios. The present study is grounded in current scientific literature and operational case studies, with the objective of assisting stakeholders in selecting and implementing suitable detection instruments in law enforcement, healthcare, and public safety operations.

## 2. Overview of Detection Techniques for Fentanyl and Its Analogues, Laboratory and Field Approaches

The chemical structure of fentanyl is modified in in illegal laboratories. This creates new variations of the substance, leading to a long list of new analogues whose structural similarity makes them difficult to detect using traditional laboratory analytical methods.

For practical purposes, detection strategies can be grouped by intended use into (i) laboratory-based confirmatory methods and (ii) field-deployable screening approaches. The former rely predominantly on mass spectrometry—typically GC–MS or LC–MS–MS, and, where available, high-resolution MS—to achieve unambiguous identification and accurate quantification of fentanyl and its analogues. The latter include portable FTIR and Raman spectrometers and rapid immunoassay/lateral-flow tests, which enable on-site triage and threat assessment. While screening tools support timely decision making, their performance can be limited by low analyte concentrations, complex mixtures and spectral interferences; positive findings should therefore be verified using confirmatory laboratory analysis. This subsection reviews the principles, strengths and limitations of both categories and outlines best practices for their complementary use in forensic, clinical and public-health contexts.

### 2.1. Gas Chromatography–Mass Spectrometry (GC–MS), Principles, Advantages, and Limitations for Detecting Fentanyl and Its Analogues

Routine forensic scientists use electron impact gas chromatography coupled with a mass spectrometer to separate and identify substances in seized drug samples [17]. The utilization of gas chromatography–mass spectrometry (GC–MS) facilitates the acquisition of two levels of discrimination for the identification of compounds. This is achieved by the generation of both the analyte retention time and a fragmentation mass spectrum. Despite the fact that mass spectrometry in combination with a separation technique provides a high level of specificity, repeatability and reproducibility, limitations still exist. A noted limitation is the time required for separation, which can range from 10 min to 30 min [18]. Analysis by GC may also require derivatization for compounds that are non-volatile, thermally unstable, and polar, which increases sample preparation time. The growing number of fentanyl analogues also presents challenges, as outdated spectral libraries can lead to misidentification or non-detection of newer compounds. These analytical challenges are often due to the nature of the samples submitted to forensic science providers for analysis. These challenges include isomeric interferences, low purity and insufficient sample amounts. Fentanyl and fentanyl-related substances are highly toxic and a significant hazard to those who ingest them. Often, fentanyl and fentanyl-related substances are mixed with other substances, resulting in fentanyl-related analytes being present in illicit samples at very low concentrations. When sample analysis includes fentanyl analogs that have the same fentanyl structure core, there is a possibility of coelution or isomeric interferences with traditional GC–MS methods. The clear discrimination of the coeluting derivatives can be achieved through the utilization of a selected ion monitoring (SIM) approach, which employs three distinct ions for each analyte [19]. Fentanyl analogs are often observed at low concentrations, which results in the respective GC–MS spectrum lacking the molecular ion [17]. GC–MS is regarded as a Category A technique in forensic science, signifying its exceptional discriminating power [20]. This designation renders a precise GC–MS result as highly reliable evidence for the identification of a compound.

Gilbert et al. presented a method for analyzing 18 fentanyl analogues in their paper. The studies were conducted on substances synthesized in laboratory conditions [19]. Samples for analysis with a concentration of 0.1 mg/mL were prepared by dissolving the substance in methanol, without any derivatization, and then injected directly into the instrument The accuracy (RSD%) of the ion ratios was calculated for three ions specific to each analyte, showing a variation between replicates of 0.3–7.7%. There was a linear response over the 2.5–25.0 µg/mL range, with satisfactory repeatability. The selected ion monitoring (SIM) mode is 100 times more sensitive than Scan Mode, the intraday precision was 100 ± 2% [19].

### 2.2. Liquid Chromatography–Mass Spectrometry (LC–MS), Application, Sensitivity, and Challenges in Biological and Seized Samples

Methods based on mass spectrometry (MS), including GC–MS, liquid chromatography tandem MS (LC–MS–MS) and liquid chromatography–high-resolution MS (LC–HRMS), have been developed to confirm the presence of fentanyl analogues or other synthetic opioids [21,22]. LC–MS (especially high-performance liquid chromatography tandem mass spectrometry, HPLC–MS–MS) has become preeminent method for fentanyl detection in biological specimens and forensic analysis. Unlike GC, liquid chromatography is an effective method for the analysis of thermally unstable substances. In the case of fentanyl and its analogues, for instance, direct injection following dilution or simple extraction is often feasible, eliminating the necessity for derivatization. This makes LC–MS well suited for complex matrices like blood, urine, or tissue, where fentanyl may be present in trace amounts amidst many other compounds. LC–MS–MS methods can detect fentanyl and dozens of analogs in a single run, with limits of detection often in the sub-ng/mL range [23,24]. The combination of chromatographic retention time and precise mass/fragmentation pattern allows confident identification even if multiple analogs are present simultaneously. For example, a toxicology lab can differentiate fentanyl, acetylfentanyl and carfentanil in a sample by their distinct masses and retention times. Like GC–MS, LC–MS is considered a Category A, highly reliable technique for drug analysis [20]. For the analysis of powder samples (e.g., seized street opioids or pharmaceutical slurries) using LC–MS–MS, the reported data is more limited compared to biological matrices. However, the sensitivity generally falls in the ng/g (ppb) range. The drawbacks of LC–MS are analogous to those of GC–MS: instruments are costly and cumbersome, necessitate expert operators, and are typically restricted to laboratory settings. Even a so-called “rapid” LC–MS analysis can require 15–30 min, in addition to the time necessary for sample preparation, thus failing to meet the criteria of immediacy. Consequently, while a police laboratory may be capable of confirming the presence of fentanyl in a pill via LC–MS within a 24-h period, an officer working at the scene is not equipped to perform LC–MS analysis independently. Furthermore, the continuous updating of methods with new reference standards is necessary to maintain broad detection for novel fentanyl analogs, which makes this a dynamic and continually evolving field [25]. Despite these challenges, LC–MS remains the gold standard for sensitivity and reliability in fentanyl detection and quantification. Current research and development endeavors are focused on miniaturizing and simplifying MS-based systems to enhance their field deployability without significant performance degradation.

Polke et al. developed and validated an LC–MS–MS method for the analysis of 25 novel synthetic opioids in hair. The method exhibited satisfactory selectivity and sensitivity, with an LOD of 0.1 pg/mg. For the majority of the analytes, imprecision was 15% [26].

### 2.3. Spectroscopic Techniques—Infrared Spectroscopy, Utility, Training, and Limitations for Community Drug Checking

Infrared (IR) absorption spectroscopy is a widely utilized instrument for the purpose of drug checking, due to its relatively low cost, the simplicity of its use, its speed of operation, the minimal sample preparation requirements it necessitates, and the availability of libraries (open source and commercial) containing thousands of drug components, including cutting agents. Recent assessments of the suitability of IR spectroscopy for community drug checking have focused on the detection and quantification of fentanyl and other compounds found in the opioid drug supply. Studies show that individuals with a basic science background can be effectively trained in the operation of IR spectrometers. Nevertheless, concerns persist. The potential for misinterpretation of data, in addition to an inability to recognize and communicate the limitations of such instruments, must be considered. Infrared (IR) absorption methods are capable of determining the most significant components (active ingredients and cutting agents) in a mixture, on the condition that each one constitutes a minimum of ≈5% of the overall mixture. It is important to note that in many cases, such a rigid rule may not be applicable. Fentanyl can be identified at low concentrations in mixtures (below the level at which reliable quantification is possible) by a small peak that appears at 705 cm^−1^ [27].

### 2.4. Spectroscopic Techniques—Raman Spectroscopy, Capabilities, Field Use, and False-Negative Rates in Fentanyl Detection

Raman spectroscopy is another type of vibrational spectroscopy that provides information about the molecular identity of a substance. However, it is based on how a molecule scatters light, as opposed to how it absorbs light in infrared spectroscopy. It is evident that Raman spectrometers generally utilize a visible light source. The measurement process can be conducted by means of transparent bags, which are characterized by their ability to prevent the absorption of visible light. As is the case with water, a number of materials that are both widely available and easily accessible, including polyethylene and glass, have been found to be weak Raman scatterers when compared to the molecular structure of most drugs. The Raman spectroscopy technique has been shown to be a rapid, non-destructive method that can be implemented with portable and robust hardware. It has the capacity to identify a wide range of opioids, cutting agents and mixtures. Colored samples (for example, opioid mixtures that have been dyed purple) present a particular challenge, as it is difficult to select a laser with a long enough excitation wavelength. Studies show that near-infrared excitation at a wavelength of 1064 nm is a successful approach for such samples, although this method is limited by its reduced sensitivity [28].

Green et al. (2020) employed Raman spectroscopy in the analysis of street-obtained samples. The findings are presumably attributable to the low purity and low volume of fentanyl detected in the selected samples that were seized [29]. The primary function of the Raman spectrometer appears to be in the field testing of samples with higher purity, such as those encountered during trafficking and transport investigations, and more generally in narcotics investigation work. In the context of ‘real-world’ drug sample testing, the Raman spectrometer demonstrated a high degree of accuracy in identifying the absence of fentanyl in samples that were subsequently confirmed to be negative by GC–MS analysis. However, the instrument also exhibited a tendency to miss a significant proportion of fentanyl-positive samples, resulting in a high false-negative rate. In the absence of a more sensitive testing step, these characteristics pose a significant challenge to the adoption of the Raman spectrometer alone for public health purposes [29].

### 2.5. Gas Chromatography–Infrared Detector (GC–IRD), Integration, Isomer Differentiation, and Analytical Advantages

Gas chromatography–infrared spectroscopy (GC–IRD) is a technique that employs the benefits of infrared spectroscopy in conjunction with the separation capabilities of gas chromatography. It can be used in conjunction with the widely utilized GC–MS technique to enhance laboratories’ capabilities when analyzing fentanyl-related substances. It can differentiate fentanyl analogs with the same molecular weight but different chemical structure. It is worthy of note that two modes are utilized for GC–IR, the vapor phase and the condensed phase. It is a acknowledged fact that vibrational frequencies are usually higher in gas-phase IR than in condensed-phase IR [17]. The GC–IRD can identify the structural isomers of novel psychoactive substances but cannot differentiate between the optical isomers. Finally, Warner et al. analyzed fentanyl analogues using GC–IRD: standard solutions at 1 mg/mL were identified with ~98% library match scores, and the retention-time coefficient of variation (CV) for each standard was <3% [30].

### 2.6. Portable Techniques, Handheld Raman and FTIR Devices for On-Site Fentanyl Identification

Raman spectroscopy and FTIR are widely adopted techniques in the rapid identification of fentanyl and other illicit substances in the field. Both methods rely on analyzing the vibrational modes of chemical bonds to generate a unique molecular fingerprint, which is then compared to spectral libraries for compound identification. Handheld devices, such as the Thermo Fisher TruNarc (Raman) and Agilent Resolve or Thermo TruDefender (FTIR), have enabled law enforcement and hazardous materials (hazmat) teams to carry out real-time analysis on-site with minimal training [2].

In optimal conditions, such as with pure or high-concentration samples, Raman and FTIR spectroscopy have been demonstrated to provide Category A-level confirmation in accordance with forensic standards [2,20].

However, these techniques have critical limitations. Their ability to detect fentanyl in mixtures is limited when the substance is present at low concentrations-typically below 5–10% of the sample weight. For example, Kimani et al. found that portable Raman and FTIR spectrometers failed to detect fentanyl in counterfeit tablets containing only 1% fentanyl, with the signal overwhelmed by excipients like paracetamol [2]. Additionally, Raman spectroscopy is affected by fluorescence, especially in dark powders or colored pills, which can obscure the spectral signal [31]. FTIR does not suffer from fluorescence but struggles with detecting compounds masked by dominant components such as sugars or fillers. Another challenge is the dependence on reference libraries. If a novel fentanyl analogue is not included, the instrument may misidentify the substance or produce no result at all. This is particularly problematic given the continuous emergence of new synthetic opioids [2,8].

To address these limitations, several advancements are being explored. One promising technique is surface-enhanced Raman spectroscopy (SERS), which utilizes metallic nanoparticles (typically gold or silver) to amplify the Raman signal. SERS has demonstrated the ability to detect fentanyl at concentrations as low as parts-per-billion [32]. Importantly, SERS should be treated as a distinct analytical workflow rather than an extension of conventional handheld Raman, employing different substrates and sample-preparation procedures and exhibiting different achievable detection limits. Liu, Zhang et al. showed that, when combined with machine learning algorithms, SERS could identify fentanyl in heroin at 1 µg/mL with over 90% accuracy and even perform in complex biological matrices such as saliva [16,23]. Although primarily a lab-based technique, portable SERS kits are under development and may eventually offer field compatibility..

Another enhancement involves RedWave Technology’s extraction kit has the potential to address the analysis of mixtures containing fentanyl and fentanyl analogues at low concentrations. This disposable field extraction kit represents a significant advancement in the field of analytical chemistry, particularly in the context of portable ATR-FTIR spectrometers. This development signifies a significant advancement in the efforts to combat the opioid epidemic, as these devices are now capable of detecting low-dose fentanyl. This kit is going to be of great importance to people working in public safety, as it will assist them in dealing with this serious health problem. The potential of artificial intelligence in this regard is also noteworthy [33].

Ti et al. demonstrated that a neural network trained on thousands of FTIR spectra could identify fentanyl with a greater degree of accuracy than human analysts [15,34]. The study achieved a 96% F1 score in detecting fentanyl in samples with less than 2% content, levels typically missed by conventional analysis.

In summary, Raman and FTIR spectroscopy are useful methods for basic identification of fentanyl in the field. Innovations such as SERS, targeted chemical extraction, and artificial intelligence (AI)-driven interpretation have the potential to enhance the capabilities of vibrational spectroscopy. This will facilitate the detection of fentanyl.

In summary, Raman and FTIR spectroscopy are valuable tools for the rapid, in-field identification of fentanyl. Conventional handheld Raman using near-infrared (NIR) excitation (e.g., 1064 nm) mitigates fluorescence interference but has lower intrinsic sensitivity, often requiring ≥1–5% *w*/*w* in mixtures for reliable detection. By contrast, surface-enhanced Raman spectroscopy (SERS) leverages plasmonic substrates to amplify Raman signals by several orders of magnitude, enabling trace- or ppb-level detection under optimized conditions. NIR Raman and SERS are therefore complementary rather than contradictory, representing distinct sensitivity regimes and hardware workflows. Further innovations such as targeted chemical extraction and AI-driven spectral interpretation hold promise for enhancing vibrational techniques in complex, real-world matrices [35,36].

### 2.7. Colorimetric Methods, Rapid, Accessible, and Presumptive Tests for Field Settings

The presence of fentanyl can be detected by colorimetric methods, which involve a reaction with assay reagents. This reaction produces a color change that can be used to analyze the substance qualitatively. The efficacy of these methodologies is evidenced by their ability to produce results in a matter of minutes, thus rendering them highly advantageous for point-of-use testing. The simplicity of these instruments and their limited instrumentation renders them accessible in field settings or environments with limited resources [37].

In line with the recommendations for rapid presumptive drug testing, Gilbert et al. used the following tests in their work [19,29]:

- The Marquis: 1% formaldehyde (37% aqueous solution) in concentrated sulfuric acid);
- Scott’s: 1% cobalt(II) thiocyanate in glycerol-deionized water (1:1, 10 mL), nitric acid (concentrated);
- Eosin: Y tests—2′,4′,5′,7′-tetrabromofluresceine in aqueous potassium phosphate buffer (pH = 7)

These were used for the presumptive detection of fentanyl and its derivatives in samples [38]. This study recommends the utilization of three presumptive tests (Eosin Y, Marquis, and Scott’s) for the discrimination of controlled drugs and/or adulterants and fentanyl derived synthetic opioids.

Mechanistically, the Marquis test is thought to proceed via acid-promoted reactions of fentanyl in concentrated sulfuric acid, yielding chromophores analogous to those observed with MDMA. In Scott’s reagent, the color change most likely arises from coordination of tertiary amines to cobalt(II) thiocyanate, driving conversion of the pink octahedral Co(II) complex to a blue tetrahedral species. The nitric-acid test is generally negative for most fentanyl derivatives; notable exceptions are 2-furanylfentanyl and 3-furanylfentanyl, which produce a yellow product, plausibly via electrophilic attack on the furan ring [19]. Eosin Y forms complexes with selected tertiary amines, giving an orange-to-pink color change; for fentanyl, binding has been reported to occur primarily at the non-piperidine ring nitrogen, with the piperidine nitrogen acting as a secondary site [39]. In cases where the results are not clear cut, the nitric acid test is to be employed as a secondary screen [19].

### 2.8. Immunoassays and Lateral-Flow Devices, Fentanyl Test Strips: Principles, Performance, and Real-World Application

LFI strips, commonly known as fentanyl test strips, are one of the simplest and most widely used tools for detecting fentanyl in the field. These small, disposable strips function like over-the-counter pregnancy tests. A small amount of drug is dissolved in water, and the strip is dipped in. Within five minutes, the appearance or disappearance of a colored line reveals whether fentanyl is present above a certain threshold. One line usually indicates a positive result, and two lines a negative though this can seem counterintuitive. The test is essentially a yes/no screening tool based on antibody interactions that detect fentanyl or some of its analogues as the sample migrates along the strip [29].

The main appeal of LFI strips is their simplicity. They are cheap (just a few dollars per strip), fast, and require no special training or equipment, making them ideal for first responders, harm reduction workers, and even the general public. Sensitivity is generally good: some strips detect fentanyl at concentrations of just a few hundred nanograms per milliliter sufficient to flag small amounts mixed into a street sample. In practical terms, that means fentanyl can be detected even if it makes up only a few percent of a powdered mixture. For example, some LFIs can detect fentanyl at low microgram levels in drug samples [29]. These strips are even used in hospitals to check for fentanyl in urine, although not all toxicology panels include it by default [40].

Despite these strengths, there are limitations. Specificity is not perfect—test strips rely on antibodies typically developed for fentanyl or one of its metabolites, meaning they may not respond equally to all analogues. For example, while a strip might detect 100 ng/mL of fentanyl, it might require up to 4000 ng/mL of carfentanil—one of the most potent analogues to trigger a positive result [41]. False negatives are possible if an uncommon analogue is not well recognized by the antibody. Conversely, false positives can occur if structurally similar non-opioids cross-react. Still, most modern strips are fairly selective and mainly cross-react with other fentanyl-class opioids.

Another issue is that these strips were originally developed for urine testing, not direct drug checking. Their use to screen street drugs is technically off-label, which introduces variability in procedures and interpretation. Instructions are now widely distributed by public health organizations to help users apply consistent protocols, but the potential for user error remains. There have also been legal challenges: in some places, fentanyl strips were initially considered drug paraphernalia. That’s changing now, with many states and countries adjusting laws to support their use due to their overdose-prevention potential [42].

Despite these caveats, the strips have proven to be lifesavers. By giving users, a quick yes/no answer, they allow for immediate decisions like discarding a batch or administering naloxone. Many public health agencies now include test strips in overdose prevention kits. Improvements are also on the horizon—including more sensitive or selective antibodies, strips that indicate high vs. low fentanyl levels, or digital readers (including smartphone apps) that can interpret results more objectively and even log data. In short, while LFIs are no substitute for lab-based confirmation, they offer a fast, affordable, and user-friendly way to screen for fentanyl and play a key role in real-world harm reduction and early intervention.

Field evaluations of commercially available LFI strips indicate sensitivity between 96–99% and specificity of 90–98% for fentanyl detection in powder samples. Precision is generally within 5–15% RSD, though it can be influenced by environmental conditions. LOQs typically fall within the low ng/mL range, and reproducibility across batches is acceptable for field screening purposes.

A significant disparity remains in the limits of detection of fentanyl and its analogs when employing immunoassays and mass spectrometry methods. In order to address this issue, Zhao et al. developed a novel immunoassay with high sensitivity for fentanyl and its analogs [43]. They used a kind of antibody that has a strong affinity for fentanyl, and fused it with luciferase (Nano-Luc-fused H23 special) to obtain a fast immunoassay that can detect fentanyl below 1 pg/mL. In their seminal paper, Yang et al. employed an atomic monocomposite, termed MnOx@Mn-MOF-5, as a quencher in a novel CLIA method for the highly sensitive detection of fentanyl [44]. The method was characterized by a detection limit of 0.03 pg/mL, a high degree of reproducibility and RSD value not exceeding 9.47%. Chemiluminescent readouts (e.g., HRP–luminol or electrode-triggered electrochemiluminescence) translate antibody binding into amplified light emission and typically deliver order-of-magnitude lower LODs than colorimetric formats while remaining compatible with microplate and automated analyzers [43,45,46]. For fentanyl, competitive CLIA with luminol signal enhancers has achieved sub-pg·mL^−1^ performance in biofluids, illustrating the sensitivity gain attainable with established immunochemical workflows [45]. Electrochemiluminescent designs (antibody- or aptamer-based) can push limits even further for fentanyl analogues in buffered samples, though matrix effects and cross-reactivity require case-by-case validation prior to broad deployment [46].

CLIA/ECLIA fit the multi-tier workflow as a highly sensitive presumptive step; presumptive positives should still be confirmed by LC–MS–MS, and calibration must address analogue-dependent cross-reactivity [43,46].

### 2.9. Electrochemical Sensors and Biosensors, Emerging Technologies for Rapid, Sensitive, and Portable Analysis

Electrochemical sensors represent an emerging class of tools for fentanyl detection that offer high sensitivity in a compact format. These sensors typically employ a working electrode (often a disposable screen-printed electrode) modified in some way to make it responsive to fentanyl’s presence. When a potential is applied, fentanyl can undergo redox reactions at the electrode surface, producing measurable electrical signals (current or voltage changes). By analyzing the voltametric profile or current peak, one can detect and even quantify fentanyl. Research in recent years has produced a variety of experimental fentanyl sensors with impressive performance [47].

One approach is to modify carbon electrodes with nanomaterials to enhance sensitivity. For example, Marenco et al. reported a disposable sensor using a screen-printed carbon electrode decorated with carboxylated carbon nanofibers [48]. Fentanyl was directly oxidized at the nanofiber-modified surface and measured via differential pulse voltammetry (DPV). The sensor showed a linear response from 0.125–10 μM and a limit of detection of ~75 nM, which is roughly 25 ng/mL—extremely sensitive for a field-deployable test [48]. Importantly, it demonstrated excellent selectivity: common cutting agents (glucose, lactose, caffeine, acetaminophen, etc.) produced no interfering signals, and even in a complex matrix (fentanyl spiked into artificial urine), the sensor performed well [48]. Such high sensitivity and matrix tolerance would allow detecting minute fentanyl levels in street samples or biological fluids within minutes.

Another strategy, by Goodchild et al., used a carbon screen-printed electrode modified with a room-temperature ionic liquid to obtain a distinctive voltametric fingerprint for fentanyl [8]. They employed cyclic square-wave voltammetry and found that fentanyl produces a signature pattern of peaks: an initial oxidation around +0.56 V, followed by a pair of reduction/oxidation peaks near −0.23 V, in a two-cycle scan. This multi-peak electrochemical signature enabled unambiguous identification of fentanyl even in the presence of other adulterants, since few substances have the same redox pattern. The assay was rapid (~1 min) and could detect micromolar concentrations of fentanyl in street-like samples [8]. The authors noted this information-rich fingerprint could help differentiate fentanyl from other opioids and that the method holds promise for “rapid decentralized fentanyl detection at the point of need”.

In general, electrochemical sensors can achieve very low detection limits (often in the tens of nM or low ng/mL range), rivalling laboratory instruments, by leveraging signal amplification from nanomaterials or redox cycling [47]. They also tend to be fast (measurement in a few minutes or less) and use inexpensive, disposable test strips. Specificity can be engineered: by tuning the electrode surface or using recognition elements (see aptamers in the next section), sensors can be made to preferentially respond to fentanyl. For example, the distinct voltametric peaks observed by Goodchild et al. serve as an intrinsic confirmation of fentanyl’s identity [8]. Cross-reactivity with other opioids is generally low if the detection relies on a unique oxidation potential or a highly selective binding element. However, one challenge is that different fentanyl analogues may have slightly different electrochemical behavior (e.g., oxidation potentials)—a sensor calibrated for fentanyl might under-respond or over-respond to certain analogs. To address this, sensors could either be designed with broad-spectrum receptors (like antibodies or aptamers that bind multiple analogs) or a panel of sensors could be used to cover various analog classes.

When applied directly to complex street samples or biological fluids, electrochemical platforms may exhibit modest signal suppression due to matrix effects. Reported decreases range from 10% to 25% in undiluted artificial urine, although electrode surface engineering and dilution can mitigate these effects without compromising detection limits. At present, most fentanyl electrochemical sensors are in the prototype or proof-of-concept stage in academic research. They show great promise for field deployment: one can envision a handheld electronic reader (similar to a glucometer) where a disposable fentanyl test strip is inserted to get an immediate reading. Indeed, the simplicity and portability of such sensors are huge advantages—a small kit could be carried by police or paramedics. Some hurdles remain, such as ensuring stability of the sensor strips, avoiding fouling of electrodes with real-world samples, and validating specificity for dozens of analogs. Nonetheless, results so far indicate that electrochemical detection could provide rapid, on-site fentanyl testing with excellent sensitivity, complementing the traditional lab-based methods [8,49,50].

### 2.10. Advancements in Fentanyl Detection Technologies, Emerging Approaches: Machine Learning, Aptamer-Based Biosensors, and Nanomaterials

The fight against fentanyl has prompted a wave of innovation, marrying advanced science and engineering to create ever more sensitive, smart, and field-friendly detection methods. In this study, we examine several emerging technologies that represent the forefront of fentanyl detection. These technologies include machine learning-enhanced spectroscopy, aptamer-based biosensors, and nanomaterial-based approaches.

***Machine Learning-Enabled SERS***

Recent advancements have demonstrated the potential of combining SERS with machine learning (ML) for the on-site detection of fentanyl. A notable study by Zhu et al. introduced a super absorbing meta surface that enhances light absorption across a broad wavelength range, achieving significant electromagnetic field enhancement [16]. This design enabled the detection of fentanyl concentrations as low as 1 μg/mL in various samples, including pure solutions, heroin mixtures, and saliva. By integrating partial least squares regression, the system predicted fentanyl concentrations with over 93% accuracy, facilitating rapid and precise detection without the need for extensive data processing or specialized personnel.

Similarly, Cooman et al. evaluated fentanyl-related compounds using electrochemical SERS (EC-SERS) combined with convolutional neural networks (CNN) [2]. Their approach achieved an overall accuracy of 98.4% in classifying fentanyl derivatives, demonstrating the efficacy of ML in enhancing the analysis of complex drug mixtures.

Additionally, a review by de Oliveira Penido et al. discussed the integration of chemometrics and ML with SERS, highlighting the improved selectivity and automation in detecting substances like fentanyl [51]. The authors emphasized that these multivariate approaches are instrumental in extracting key information from complex datasets, thereby advancing the development of automated SERS sensors for real-world applications.

These studies collectively underscore the promise of ML-enabled SERS in providing rapid, accurate, and portable solutions for fentanyl detection, addressing critical needs in public health and law enforcement.

***Aptamer-Based Biosensors***

Another emerging strategy employs aptamers—short DNA or RNA oligonucleotides that can fold into shapes that bind specific target molecules (analogous to antibodies but made of nucleic acids). Aptamers can be selected in vitro to have high affinity and specificity for fentanyl. Once obtained, they can be integrated into various sensing platforms (electrochemical, fluorescence, colorimetric, etc.) to create fentanyl detectors. Aptamers offer some advantages over traditional antibodies: they are synthetically produced (no animals needed), relatively stable, and can be readily modified with tags for sensing. A recent study by Canoura et al. used SELEX (Systematic Evolution of Ligands by EXponential enrichment) to isolate new DNA aptamers that bind fentanyl with nanomolar affinity [52,53]. The selected aptamers showed excellent specificity, with minimal binding to dozens of potential interferents (including other drugs and common metabolites). The authors then incorporated one of these aptamers into an electrochemical aptamer-based (E-AB) sensor. In their design, the aptamer was attached to an electrode and tagged such that when fentanyl binds, it causes a measurable electrical signal change. The sensor was able to rapidly detect fentanyl in complex biological fluids—they demonstrated detection in 50% diluted serum, urine, and saliva at clinically relevant concentrations [52].

The performance reported is impressive: the aptamer sensor had a limit of detection around 10 nM (≈3–4 ng/mL) for fentanyl in buffer, and similarly low detection limits in diluted human fluids. This is sensitive enough to cover the range of interest for both therapeutic monitoring and many overdose cases. Moreover, it maintained specificity: tests showed <10% cross-reactivity to a panel of 22 different interferent compounds (including other opioids, stimulants, and common cutting agents) even when those were present at concentrations 5–10 times higher than fentanyl. In other words, the aptamer sensor largely ignores other substances and responds chiefly to fentanyl, which is exactly what you want to avoid false positives. The response was also rapid (on the order of seconds to a minute). Given these attributes, aptamer-based sensors could be developed into point-of-care devices for scenarios like emergency rooms (to test overdose patients) or law enforcement (field testing of powders).

Aptamers can also be used in non-electrochemical formats. For instance, one could design a lateral-flow strip with aptamer-based detection instead of antibodies, potentially improving stability and shelf-life. Or aptamers could be coupled with fluorescent reporters to make a simple mix-and-read test (similar to some DNA testing kits). While these ideas are in early stages for fentanyl, the successes so far indicate a strong potential. Aptamers are sometimes dubbed “chemical antibodies”—they mimic the binding specificity of antibodies but are easier to produce and modify. As such, they are well suited to detecting small molecules like fentanyl, which can be challenging for traditional immunoassays. Future aptamer sensors might achieve even lower detection limits (through signal amplification schemes) and broader analog coverage (perhaps by using multiple aptamers in one device). The 2023 study is a proof of concept that showcases aptamers as a viable path to rapid, sensitive, and specific fentanyl detection, particularly in biomedical contexts where the sample matrix is complex [54,55]. Aptamer-based platforms demonstrate strong selectivity even in complex matrices, but validation studies indicate a measurable signal reduction (5–8%) in diluted serum compared to buffer, underscoring the importance of calibrating such devices for realistic sample conditions.

***Nanomaterials and Other Novel Approaches***

Beyond SERS and aptamers, a variety of other novel techniques are being explored. Many involve nanomaterials to improve performance. For example, metal–organic frameworks (MOFs) and various nanoparticles have been used to modify electrodes or as part of optical sensors. One study constructed an electrochemical sensor with a Zn-based MOF coating a carbon electrode; the porous MOF enriched fentanyl from solution and catalyzed its oxidation, achieving a detection limit around 0.3 μM. The utilization of this electrode type confers several additional advantages, including cost-effectiveness, efficiency, and ease of use. Furthermore, the screen-printed design allows for its use as a single-use sensor, thus helping to prevent cross-contamination [47]. The sensor could successfully detect low levels of fentanyl spiked in urine and plasma.

Another concept is using molecularly imprinted polymers (MIPs). The purpose of these synthetic materials is to provide specific locations for target molecules to bind. This results in a state of interconnection that can be likened to that of a lock and key. MIPs differ from biological receptors such as antibodies, aptamers and enzymes. In conditions of extremity, these objects have been shown to exhibit enhanced stability, increased resistance to breakdown, and the capacity for multiple reuse. Liu et al. constructed a special sensor known as a long-period fiber grating (LPFG) sensor [56]. The sensor was augmented with nanoMIPs, special tiny particles. The fabrication of these particles was achieved through a process designated as solid-phase imprinting. The test was designed to detect a substance known as carboxyl-fentanyl. This finding signified a modification in the manner in which light bounced off the surface, thereby enabling the test to detect minimal quantities (50 ng/mL) of the substance with exceptional precision in bodily fluids.

Additionally, IMS a technology widely used for explosive detection, has been investigated for fentanyl. IMS is capable of detecting trace vapors or particles by observing the drift of ions in an electric field. This process is rapid, taking only seconds, and portable units are available for use. However, the low vapor pressure of fentanyl results in its poor detectability by the IMS. Preheating or thermal desorption can be employed as a means of liberating a sufficient quantity of analyte for detection. The resolving power of portable IMS instruments is typically in the range of R ≈ 30–60, where *R* is defined as the ion drift time divided by the peak width at half maximum (FWHM). This moderate resolution can complicate the distinction between fentanyl and closely related compounds such as carfentanil, remifentanil, methadone, and common cutting agents [57]. False-positive results may occur when ion drift times overlap, emphasizing the importance of confirmatory analysis with higher-specificity techniques.

In summary, a range of emerging technologies are on the horizon. Many of these aim to combine high sensitivity/specificity with field portability—essentially to bring laboratory-level detection to non-lab settings. Table-top mass spectrometers (e.g., a DART–MS), hybrid devices that combine a presumptive test with a confirmatory technique, smartphone-based analyzers, and even drones or robots with on-board detectors for hazardous scenes are being discussed [28]. While not all of these will come to fruition, the intense focus on the fentanyl problem is accelerating innovation. The next generation of fentanyl detection tools will likely incorporate multimodal approaches (e.g., an instrument that does an optical scan *and* an electrochemical test in parallel) and smart data analysis (AI algorithms to interpret results and cross-reference drug libraries). These emerging solutions, once matured and validated, should greatly enhance our ability to quickly and accurately detect fentanyl and its myriad analogues in any scenario.

To provide a clearer and more objective view of the maturity and deployment potential of emerging fentanyl detection technologies, Table 1 summarizes their estimated Technology Readiness Levels (TRLs), current regulatory validation status, main commercialization barriers, and contextual notes on performance claims. The table highlights that most novel platforms, including aptamer-based biosensors, nanoMIP-LPFG systems, ML-enabled SERS, and portable electrochemical aptasensors, are still in early prototype stages (TRL 2–5) and have yet to undergo formal regulatory approval. While the reported analytical performance in laboratory studies is promising, further independent, multi-site validation in realistic operational conditions will be essential for advancing these methods towards field readiness.

## 3. Comparison of Detection Methods

Each detection modality has its own strengths and weaknesses. As demonstrated in Table 2 and Table 3, a summary of key comparisons is provided in relation to the limit of detection and the advantages and limitations of the selected methods.

**Matrix effects and their impact on detection performance**

The analytical performance of fentanyl detection technologies can be substantially influenced by matrix effects, which occur when components other than the target analyte alter the analytical signal. In biological fluids such as blood, urine, or saliva, proteins, salts, and endogenous metabolites may cause suppression or enhancement of signals, while in seized street samples, the presence of cutting agents (e.g., lactose, caffeine, sugars) or other active drugs can interfere with target recognition.

Laboratory-based techniques (e.g., GC–MS, LC–MS) typically address matrix effects through sample preparation steps such as solid-phase extraction, liquid–liquid extraction, or derivatization, achieving recoveries >95% and minimal signal suppression under validated conditions. In contrast, field-deployable methods—especially electrochemical and biosensing platforms—often require minimal or no sample preparation, making them more susceptible to matrix-related performance degradation [59].

Quantitative evaluations indicate that for electrochemical sensors, matrix interference can reduce fentanyl peak currents by 10–25% in undiluted artificial urine, depending on the electrode modification. For example, Marenco et al. reported recovery rates above 90% in 50% diluted urine, whereas full-strength urine caused an ~18% decrease in signal compared to buffer [48]. Aptamer-based electrochemical biosensors developed by Canoura et al. maintained <10% cross-reactivity with 22 tested interferents, yet showed a 5–8% decrease in signal amplitude in diluted serum relative to buffer [52].

Complex drug mixtures introduce additional challenges. Electrochemically inactive excipients like lactose typically do not interfere, but high concentrations of redox-active substances (e.g., ascorbic acid) can generate background peaks or partially mask fentanyl’s oxidation signal. Goodchild et al. observed that in heroin–fentanyl mixtures, the characteristic oxidation peak of fentanyl (+0.56 V) was still detectable but reduced in height by ~12% when heroin content exceeded 80% by mass [8].

These data highlight the need for validation of detection technologies under realistic operational conditions. Potential mitigation strategies include pre-filtration, selective extraction, and electrode surface modifications with antifouling coatings, which can improve robustness in complex matrices.

Laboratory-based methods such as GC–MS and LC–MS–MS generally demonstrate high accuracy (>95%), low intra-day variability (<5% RSD), and excellent inter-laboratory reproducibility, with LOQs in the low-ng/mL or sub-ng/mL range. Field-deployable technologies such as LFI strips or portable spectrometers show greater variability, with reported precision often in the 5–15% range and performance more susceptible to matrix and environmental effects.

**Table 2:** Comparison of fentanyl detection methods used in laboratory.

Analytical Method	Limit of Detection	Advantages	Limitations	Ref.
GC–MS	~0.1–1 ng (solid samples or extracts)	high sensitivity, high specificity, widely accepted, established libraries	cost and training, carrier gas dependency, aplicable to substances that are thermally stable	[2]
LC–MS	0.25–5 ng/mL	great for low-level detection, high sensitivity, can detect fentanyl in complex mixtures	required well-prepared personnel and lab infrastructure	[60]
FTIR	~10% *w*/*w*	non-destructive, rapid method which does not require complicated sample preparation	matrix interferences’ which resulted in low sensitivity, library matching	[61]
GC–IR	~10–100 ng per injection	structural confirmation, high chemical specificity	less sensitive than MS; slow throughput; bulkier instruments; limited portability	[2]

## 4. Future Directions and Regulatory Considerations in Fentanyl Detection

The evolving fentanyl crisis demands both scientific innovation and responsive public policy. Advancements in detection technology have been primarily focused on enhancing the comprehensiveness, portability, and user-friendliness of the tools, with a particular orientation towards their utilization in field settings by non-specialists (see Table 3 for a comparison of field-deployable fentanyl detection methods). A significant challenge is posed by the rapidly emerging novel fentanyl analogues, which are designed to evade detection by current testing methods. To address this, researchers are developing multiplexed sensors combining different recognition elements, such as antibodies, aptamers, or molecularly imprinted polymers to improve broad-spectrum detection [62]. Hybrid platforms that integrate rapid immunoassays with confirmatory techniques like Raman spectroscopy or SERS are also under development, offering both speed and specificity in a single tool [63].

**Table 3:** Comparison of fentanyl detection methods used in field.

Analytical Method	Limit of Detection	Advantages	Limitations	Ref.
Colorimetric Tests/Lateral-Flow Assays	~10–100 ng/mL (varies by reagent/test)	cheap, easy to use, rapid and ready for use in the field.	possible false positives/negatives, cannot differentiate analogs, low specificity,	[64,65]
Handheld FTIR	~1–5% *w*/*w* (~10,000 ppm)	non-destructive, fast, can identify cutting agents	poor for low-concentration samples or mixtures	[2,29]
Handheld Raman	~1–10% *w*/*w*; SERS: ~0.1–10 ng/mL	non-contact, library matching, SERS gives trace detection	fluorescence interference (Raman), poor reproducibility (SERS)	[66,67]
Electrochemical Sensors/Aptamer Sensors	~1–100 ng/mL (some aptamer sensors reach 10 nM)	low-cost, rapid, miniaturizable, promising for analogs	still emerging; affected by sample matrices	[58,68]
Immunoassay Strips	~1–10 ng/mL	very rapid, simple, portable, selective to fentanyl and some analogs	limited quantification, potential cross-reactivity	[13,69]

Miniaturization represents a significant driving force. Technologies that were previously confined to laboratory settings, such as mass spectrometry, SERS, and even gas chromatography, are being adapted into portable formats. The present study investigates the testing phase of prototypes of smartphone-based colorimetric analyzers and field-deployable gas chromatography–mass spectrometry systems [70]. These devices, when paired with regularly updated spectral libraries, could enable real-time identification of novel fentanyl analogy directly at points of interdiction or overdose scenes.

At a broader level, global cooperation is essential. This includes regulating precursor chemicals, sharing reference materials, and monitoring wastewater for opioid residues to track trends [71].

To speed deployment, regulatory pathways for innovative devices should be streamlined. Emergency use authorizations or fast-track FDA approvals may help deploy diagnostics more quickly, particularly in public health or forensic settings. Agencies like NIST and SWGDRUG can assist by setting performance standards to ensure field results hold up in court [72].

Ultimately, tackling fentanyl requires a multidisciplinary response: smarter technology, informed regulation, and strong inter-agency cooperation. Equipping frontline workers with effective tools will be key to staying ahead of this fast-moving threat.

**Hazardous Materials (HazMat) Response and CBRN Incidents**

Fentanyl detection has become integral to hazardous materials (HazMat) and CBRN incident response. Responders increasingly encounter fentanyl in diverse scenarios ranging from accidental spills to deliberate releases. Field-deployable FTIR, Raman, and IMS instruments are standard equipment for HazMat teams, identifying opioids and other chemical hazards on-site [2].

In the context of CBRN threats, the potential of fentanyl as a chemical weapon or incapacitating agent has been recognized, with relevant agencies such as the FBI, the Department of Homeland Security and Organisation for the Prohibition of Chemical Weapons taking note. It is an increasingly prevalent feature of CBRN drills to incorporate fentanyl in order to assess the readiness of responders. Exercises conducted by the military and civilian sectors test detection technologies in conjunction with standard chemical and biological threats, thereby reinforcing preparedness. It has been posited that fentanyl should be categorized as a weapon of mass destruction (WMD) on account of its potency and lethal potential in scenarios involving mass exposure. This would necessitate the development of enhanced detection and response strategies [73].

Unexpected encounters with fentanyl in domestic or public settings require responders to carry portable detectors and naloxone for safety. Hazardous device teams incorporate fentanyl detection into procedures to protect against booby-traps or intentional contaminations. Tactical protocols dictate immediate use of FTIR or Raman devices to evaluate hazards and guide protective actions such as containment and evacuation [2].

In the event of large-scale incidents, the deployment of mobile laboratories equipped with GC–MS has been shown to facilitate ongoing support for the detection and identification of problems, thereby ensuring a response that is both informed and effective. The incorporation of fentanyl detection into security measures and HazMat/CBRN responses has been demonstrated to enhance safety, efficiency, and preparedness [2].

## 5. Conclusions

Fentanyl and its analogues continue to drive demand for rapid, reliable detection across laboratory and field settings. This review synthesizes current capabilities spanning confirmatory mass spectrometry (GC–MS, LC–MS–MS), widely deployed vibrational tools (FTIR, handheld Raman), presumptive assays (colorimetric and lateral-flow immunoassay strips), and emerging approaches (SERS, electrochemical and aptamer-based sensors, and ML-enabled analytics). Together, these technologies form complementary layers: field screening to triage risk, followed by laboratory confirmation for definitive identification and quantification. The integration of hybrid and advanced technologies has the potential to resolve existing limitations and deliver laboratory-quality results in real time in the near future.

Notwithstanding, it is crucial to recognize that a single solution, whether technological or methodological, will not suffice to address the fentanyl crisis in its entirety. A comprehensive approach, integrating a range of methods in a complementary manner, is essential. This approach involves the use of test strips for screening, portable spectrometers for identification, and laboratory tests for confirmation and quantification. Despite substantial progress, important limitations remain. Handheld FTIR and Raman deliver speed and non-destructive analysis but typically require ≥1–5% *w*/*w* in mixtures and depend on library coverage; fluorescence and dominant excipients can obscure signals. SERS achieves trace/ppb-level detection under optimized conditions, yet performance can vary with substrate quality, preparation, and complex matrices. LFI strips are inexpensive and simple, but may exhibit cross-reactivity, analogue-dependent sensitivity, and off-label variability for direct drug checking. Electrochemical and aptamer platforms show low LODs and fast response, but most are at early TRLs, with open challenges in long-term stability, antifouling, and broad analogue coverage. Even for MS, portability, sample prep, and continual library/method updates constrain real-time field deployment. Across platforms, matrix effects (biological fluids, street mixtures) can suppress or distort signals and must be quantified and mitigated to ensure operational reliability. To translate promise into practice, three priorities stand out: (i) adopt standardized performance benchmarks—LOD/LOQ, accuracy, precision (RSD), S/N, robustness to defined matrix panels, and inter-laboratory reproducibility—reported consistently across studies; (ii) invest in multi-site, real-world validation and clear regulatory pathways (e.g., alignment with SWGDRUG; where relevant, FDA/CE), supported by open spectral and electrochemical reference libraries that track novel analogues; and (iii) accelerate engineering for deployment, including ruggedized hardware, stable recognition elements (aptamers/MIPs), simple extraction workflows for mixtures, and interpretable ML models that generalize across instruments and conditions. Chemiluminescent immunoassays (CLIA/ECLIA) offer pg·mL^−1^-level sensitivity on accessible platforms, complementing on-site screening and laboratory confirmation across clinical, forensic, and public-health contexts. By extending the immunoassay tier with chemiluminescent readouts, detection workflows gain an additional layer of sensitivity that bridges simple lateral-flow strips and confirmatory LC–MS–MS, further reinforcing the multi-tiered approach outlined in this review. This enhanced approach will directly contribute to the preservation of lives, as rapid and precise identification of fentanyl enables the dissemination of timely warnings, the provision of more effective medical treatment for overdoses, and the more efficient removal of fentanyl from circulation. The profound societal devastation wrought by fentanyl underscores the imperative for sustained innovation in detection technologies. By allocating resources to the development of these tools and implementing policies that support their utilization, public health and safety officials can enhance their capacity to respond to the opioid epidemic. Progress along these axes should yield field-ready systems with laboratory-grade confidence, improving safety for first responders and the public while supporting evidentiary standards.

**Declaration of generative AI and AI-assisted technologies in the writing process**

During the preparation of this work the author used AI to improve language and readability. After using these tools/services, the author reviewed and edited the content as needed and takes full responsibility for the content of the publication.

## Figures and Tables

**Table 1:** Technology maturity and adoption barriers for selected novel fentanyl detection methods.

Technology	TRL *	Regulatory Validation Status	Main Commercialization Barriers	Notes on Performance Claims	Ref.
Aptamer- based electrochemical biosensors	3–4	No FDA/CE/SWGDRUG approval; single-lab validation only	Aptamer stability, high reproducibility, integration into portable devices	High specificity/sensitivity reported in controlled single-lab studies; requires independent multi-site validation	[48,58]
nanoMIP-LPFG systems	2–3	No regulatory validation	Complexity of nanoMIP synthesis, robust attachment to LPFG under field conditions, lack of standardized criteria	Promising sensitivity demonstrated in laboratory conditions; untested in real-world matrices	[52,57]
ML-enabled SERS	3–4	No regulatory validation	Cost/durability of SERS substrates, generalization of the algorithm to new analogues, durability of portable devices	High classification accuracy in single-lab datasets; algorithm performance in operational environments unverified	[15,47]
Portable electrochemical aptasensors (field prototypes)	4–5	No formal regulatory approval; limited pilot studies	Device durability, user training, battery life, interference caused by complex matrices	Low-LOD detection achieved in lab and limited field trials; broader matrix validation needed	[42,50]

* TRL according to EU/US definitions: 1 = basic principles observed, 9 = system proven in operational environment.

## Data Availability

No new data were created or analyzed in this study.

## Abbreviations

The following abbreviations are used in this manuscript:

CBRN	Chemical, Biological, Radical, Nuclear incident
CNN	Convolutional Neural Networks
DPV	Differential Pulse Voltammetry
E-AB	Electrochemical Aptamer-Based
EC-SERS	ElectroChemical SERS
FDA	Food and Drug Administration
FTIR	Fourier-Transform Infrared Spectroscopy
GC–IRD	Gas Chromatography–Infrared Spectroscopy
GC–MS	Gas Chromatography coupled with Mass Spectrometry
HPLC–MS–MS	High-Performance Liquid Chromatography tandem Mass Spectrometry
IMS	Ion Mobility Spectrometers
INCB	International Narcotics Control Board
IR	Infrared Absorption spectroscopy
LC–HRMS	Liquid Chromatography–High-Resolution MS
LC–MS	Liquid Chromatography coupled with Mass Spectrometry
LC–MS–MS	Liquid Chromatography tandem MS
LFI	Lateral-Flow Immunoassays
LPFG	Long-Period Fiber Grating
MIPs	Molecularly Imprinted Polymers
ML	Machine Learning
MOFs	Metal–Organic Frameworks
MS	Mass Spectrometry
SELEX	Systematic Evolution of Ligands by EXponential enrichment
SERS	Surface-Enhanced Raman Spectroscopy
SIM	Selected Ion Monitoring
SWGDRUG	Scientific Working Group for the Analysis of Seized Drugs
UNODC	United Nations Office on Drugs and Crime
